# *Plasmodium falciparum* histidine-rich protein II causes vascular leakage and exacerbates experimental cerebral malaria in mice

**DOI:** 10.1371/journal.pone.0177142

**Published:** 2017-05-05

**Authors:** Priya Pal, Amanda E. Balaban, Michael S. Diamond, Photini Sinnis, Robyn S. Klein, Daniel E. Goldberg

**Affiliations:** 1 Department of Medicine, Division of Infectious Diseases, Washington University School of Medicine, St Louis, Missouri, United States of America; 2 Department of Molecular Microbiology, Washington University School of Medicine, St Louis, Missouri, United States of America; 3 Department of Molecular Microbiology and Immunology, Johns Hopkins Bloomberg School of Public Health, Baltimore, Maryland, United States of America; 4 Johns Hopkins Malaria Research Institute, Johns Hopkins Bloomberg School of Public Health, Baltimore, Maryland, United States of America; 5 Department of Pathology and Immunology, Washington University School of Medicine, St Louis, Missouri, United States of America; 6 Department of Neurobiology, Washington University School of Medicine, St Louis, Missouri, United States of America; Université Pierre et Marie Curie, FRANCE

## Abstract

A devastating complication of *Plasmodium falciparum* infection is cerebral malaria, in which vascular leakage and cerebral swelling lead to coma and often death. *P*. *falciparum* produces a protein called histidine-rich protein II (HRPII) that accumulates to high levels in the bloodstream of patients and serves as a diagnostic and prognostic marker for falciparum malaria. Using a human cerebral microvascular endothelial barrier model, we previously found that HRPII activates the endothelial cell inflammasome, resulting in decreased integrity of tight junctions and increased endothelial barrier permeability. Here, we report that intravenous administration of HRPII induced blood-brain barrier leakage in uninfected mice. Furthermore, HRPII infusion in *P*. *berghei-*infected mice increased early mortality from experimental cerebral malaria. These data support the hypothesis that HRPII is a virulence factor that contributes to cerebral malaria by compromising the integrity of the blood-brain barrier.

## Introduction

Malaria is a disease that afflicts several hundred million people each year. Most of the estimated 600,000 deaths [1] are due to the species *Plasmodium falciparum*, which can cause complications such as severe anemia, respiratory distress and cerebral malaria (CM). CM manifests with a progression of symptoms from decreased consciousness to coma and death. Cerebral edema due to blood-brain barrier (BBB) compromise ultimately results in brain herniation and death [2].

Infection of mice with the rodent malaria parasite strain *P*. *berghei ANKA* serves as a small animal model for cerebral malaria. The pathology present in experimental cerebral malaria (ECM) is similar to that in human cerebral malaria (CM) with notable exceptions being limited sequestration of infected RBC and a more robust immune response enriched in leukocytes [3–5]. The biological basis of these differences is controversial and poorly defined [6–9].

Histidine-rich protein II (HRPII) is a protein produced by *P*. *falciparum* but not by other malaria parasite species. It accumulates in the bloodstream [10] and is the basis of diagnostic and prognostic tests for falciparum malaria. Our previous work has shown that HRPII, at concentrations seen in patient plasma, can disrupt human cerebral microvascular endothelial cell barriers by triggering the endothelial cell inflammasome [11]. This sets off a signaling pathway that causes cell-cell junctional protein rearrangement and decreased barrier resistance [11].

In the current study, we evaluated the effect of HRPII infusion in mice. We find that HRPII causes cerebral vascular leakage in uninfected mice and increases the incidence of early death in a rodent malaria model. Blockade of inflammasome signaling with anti-IL1β antibody mitigates cerebral barrier compromise. These data support the hypothesis that HRPII contributes to the pathogenesis of cerebral malaria.

## Materials and methods

### Antibodies

Armenian hamster anti- mouse IL-1β was purchased (Leinco, I-437) and used for *in vivo* studies along with an Armenian hamster IgG isotype control (Leinco, I-140), at 300 μg/mouse. Dilutions were made in PBS.

### HRPII purification

The coding sequence for the mature form of HRPII was cloned into pET-15b (Novagen) without a tag, expressed and purified from *E*. *coli* lysate using nickel-affinity chromatography as described (27). Protein was exchanged into 20 mM Tris, 500 mM NaCl, 50 mM imidazole and loaded on a 5 ml nickel column (GE Healthcare). After washing with 60 column volumes of 20 mM Tris, 10 mM NaCl, 0.1% Triton X-114 to remove residual LPS, the column was washed with 20 column volumes of loading buffer and eluted with loading buffer with 1 M imidazole. All preparations of HRPII were tested for residual LPS using a LAL endotoxin test (Charles Rivers, R1708K); levels administered to mice contained less than 5 EU/kg. Fully active preparations of the protein were used for experiments. Activity was measured using a Factor Xa assay [12]. Protein concentration was determined by BCA assay (Fisher).

### Mouse model of cerebral malaria

Four-week old female C57BL/6 mice were purchased from Taconic. Animals were housed under pathogen-free conditions. All experiments were approved by and performed in compliance with Animal Studies guidelines at Washington University and Johns Hopkins University. Mice were given retro-orbital intravenous injections (50 μg of recombinant HRPII or BSA, in 100 μl of PBS) approximately 12 hours prior to infection. The mice were infected by retro-orbital inoculation of *P*. *berghei* ANKA parasites (10^5^ parasites/100 μl) derived from stock mice (Swiss-Webster, Taconic) with parasitemias of <3%. All efforts were made to minimize animal suffering. We needed to use early death (6–9 days) as an endpoint rather than euthanization of ill-looking animals because the mice are capable of recovering from infection and it was important to distinguish those that recover from those that do not. Animals that survived the early period (30–70%) were euthanized for high parasitemia or sick appearance, using ketamine/xyazine overdose.

### *In vivo* assessment of BBB permeability

Two doses of recombinant HRPII (200 μg in 100 ul) were given 24 hours apart by retro-orbital injection to 4-week old female C57BL/6J mice from Jackson Labs. 48 hours post initial injection, the mice were evaluated for sodium fluorescein extravasation as previously described [13]. In some mice, anti-IL-1β antibody or isotype control (300 ug each) was administered with the first HRPII infusion.

### Statistical analysis

All data were analyzed using Graph Pad Prism 6.0 (Graph Pad Software, La Jolla, CA, USA). A p<0.05 was designated as significant. Pair-wise comparisons were analyzed by two-tailed t-test. Log rank test was used to compare survival between the groups in the Kaplan-Meier survival curves.

## Results

### HRPII promotes increased BBB permeability *in vivo*

**Fig 1:**
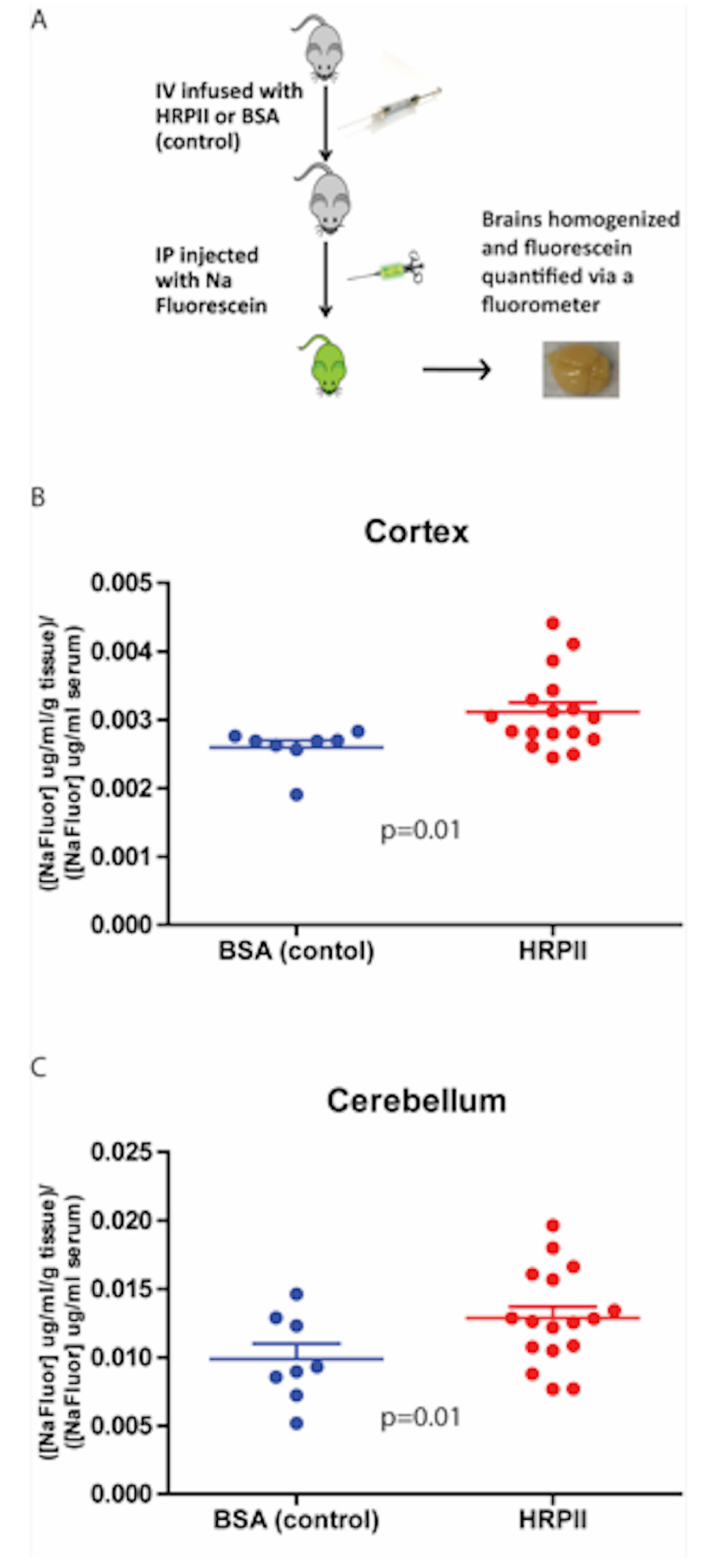
HRPII causes vascular leakage *in vivo*. **(A)** Scheme of experimental design. Two doses of HRPII or BSA (200 μg) were injected into 4-week old female C57Bl/6 mice at 0 and 24 hours. At 48 hours, fluorescein levels in the cortex (**B**) and cerebellum (**C**) of the mice was measured. HRPII treatment was significantly different from control by two-tailed t-test, p = 0.01 (cortex) and p = 0.02 (cerebellum). Data are mean values +/-SEM for 8–16 mice per group accumulated over 3 independent experiments.

In previous work, we showed that HRPII can disrupt a human cerebral endothelial cell barrier *in vitro* [11]. At concentrations found in patients with cerebral malaria [14], HRPII triggered inflammasome activation, resulting in junctional protein rearrangement and barrier leakage. To determine whether the HRPII could induce a compromise in barrier integrity of the brain endothelium *in vivo*, uninfected mice were administered two 200 μg doses of HRPII 24 hours apart by intravenous (IV) injection, and fluorescein extravasation was measured in the brain parenchyma (Fig 1A). We observed an increase in vascular leakage of fluorescein into the cortex and cerebellum of mice infused with HRPII compared to control animals (Fig 1B and 1C). Peak serum HRPII levels at one-hour post infusion were 300–400 ng/ml. At the time of harvest, HRPII levels were 150–200 ng/ml.

**Fig 2:**
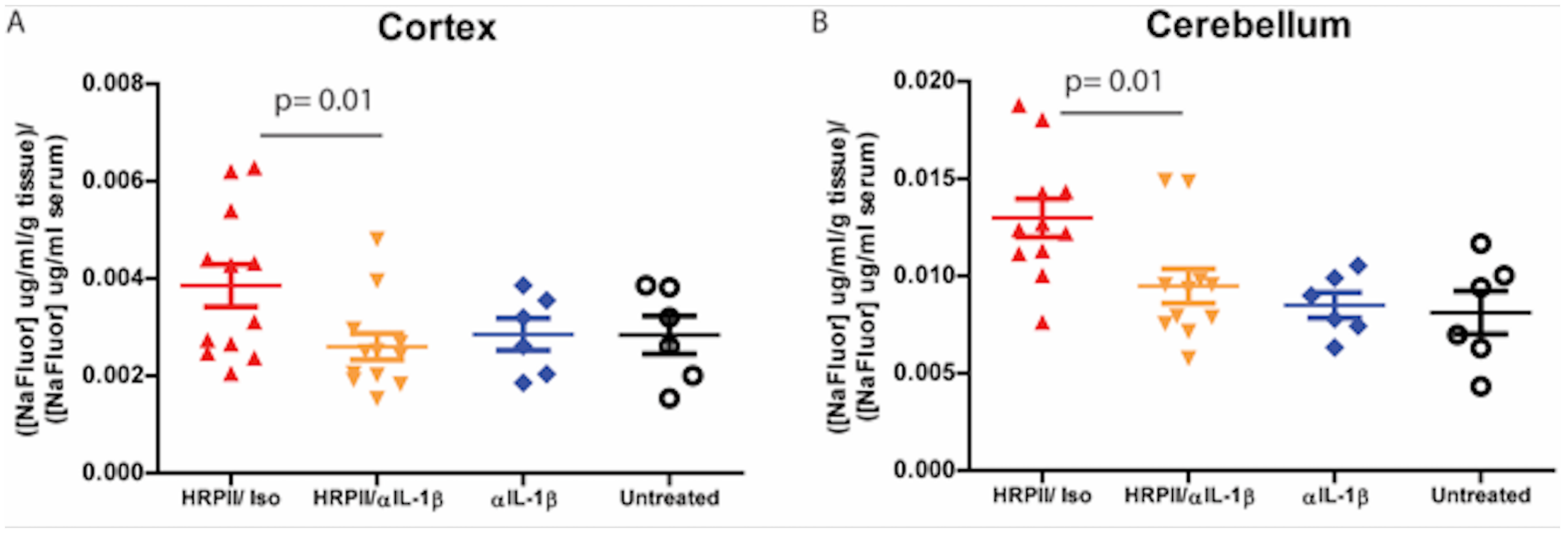
HRPII-mediated vascular leakag*e* is blocked by antibody to IL-1β. Mice were infused as in Fig 1, with HRPII plus an isotype antibody (Iso, positive control), HRPII plus anti-IL-1β antibody (experimental condition) or anti-IL-1β antibody alone (negative control). Untreated mice (no HRPII, no antibody) served as a further control. Vascular leakage in mice infused with HRPII/isotype is statistically significantly different from mice infused with HRPII/ anti-IL-1β, p = 0.01 (cerebellum) and p = 0.01 (cortex), by two-tailed t-test; p = 0.003 (cerebellum) and p = 0.06 (cortex) by ANOVA one-way variance with significance between HRPII/isotype and HRPII/ anti-IL-1β. Data are mean values +/-SEM for 6–12 mice per group accumulated over 3 independent experiments.

Our *in vitro* BBB model suggested that HRPII-mediated permeability was inflammasome dependent. To assess this effect *in vivo*, we infused uninfected mice with a neutralizing antibody to IL-1β or an isotype control and then treated them with either HRPII or a control protein (Fig 2). IL-1β-specific antibody blocked HRPII-induced sodium fluorescein leakage but did not affect sodium fluorescein extravasation in animals not receiving HRPII. These data confirm the *in vivo* relevance of IL-1β-mediated signaling for the actions of HRPII on BBB permeability.

### HRPII reduces host survival in an experimental cerebral malaria model

**Fig 3:**
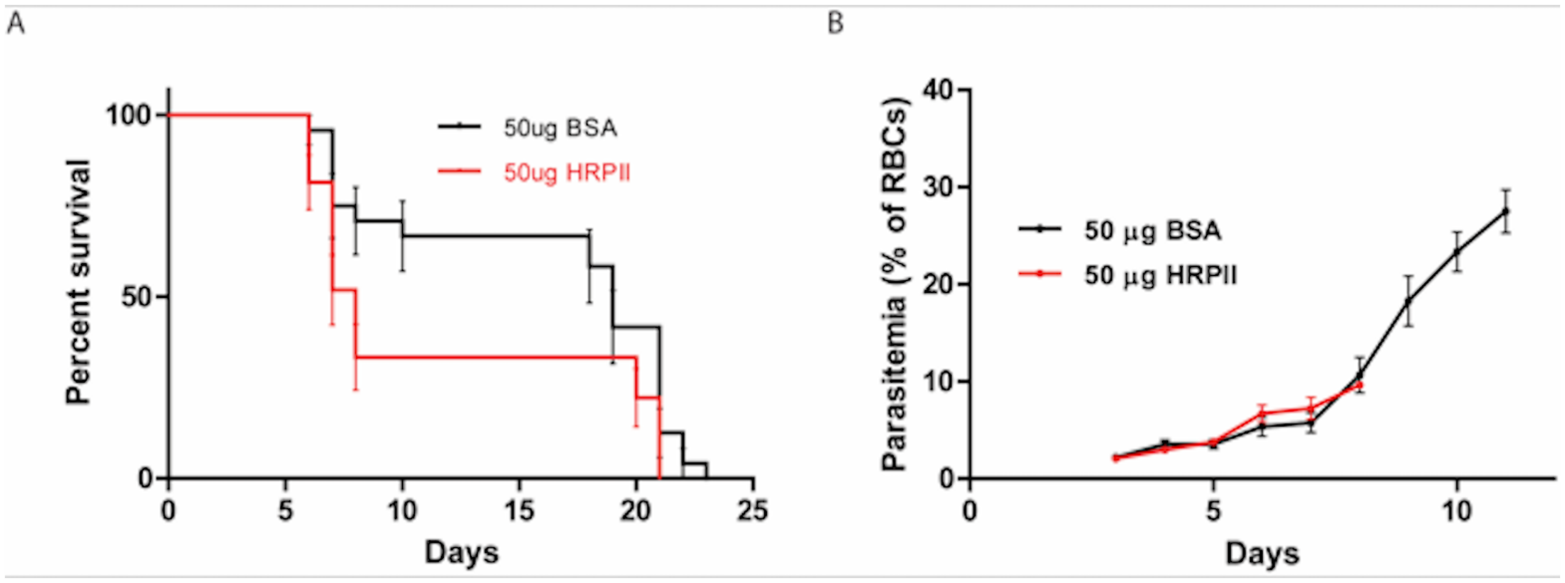
HRPII reduces survival time in an experimental cerebral malaria model. (**A**) Survival curves of 4-week old female mice infused with 50 μg of BSA or HRPII prior to infection with *P*. *berghei* ANKA (10^5^ parasites). Shown are the means for n = 24 to 27 mice pooled from four independent experiments. Curves are significantly different, p = 0.03, by the log-rank (Mantel-Cox) test. Mean time to death for HRPII = 11.5 days and for BSA = 16 days, p = 0.018, by two tailed t-test. (**B**) Mice displaying cerebral malaria-like symptoms died at low parasitemia by day 10, yet parasitemias between HRPII-infused mice and controls were closely matched on each day. Representative data from one of three experiments shown in panel A, 10 mice per group.

We next determined whether the compromise in vascular integrity observed with purified HRPII had clinical consequences during malaria infection in mice. We infused 6-week old female C57BL/6 mice with 50 μg of BSA or HRPII prior to infection with 2 x10^5^ *P*. *berghei ANKA* infected erythrocytes. Experimental cerebral malaria in mice infected with *P*. *berghei ANKA* has variable penetrance, with a 40 to 100% lethality rate from cerebral malaria [8,15] defined as neurological symptoms and death at or below 10% parasitemia, by day 10 post infection. Mice infused with HRPII had early lethality compared to control mice (Fig 3; mean time to death for HRPII = 10 days and for BSA = 16 days, *P* = 0.018). The parasitemia of mice dying from cerebral malaria-like symptoms was low, as expected, and importantly, no differences in parasitemia were observed between mice infused with HRPII or control protein.

## Discussion

BBB integrity disruption during *Plasmodium falciparum* infection is a hallmark of CM. Our previous studies identified HRPII as a parasite virulence factor that activates host endothelial innate immunity through an inflammasome-mediated pathway. This causes redistribution of endothelial junctional proteins, and increased BBB permeability.

In the current work, we found that HRPII is active *in vivo*, resulting in leakage of fluorescein into the brain parenchyma of uninfected mice as well as an exacerbation of experimental CM when the protein is infused into mice prior to infection. Mouse endothelial cells are less sensitive to HRPII than human cells (not shown) and achievable plasma levels of HRPII are considerably lower than those seen in human patients with cerebral malaria, where levels of 1–100 μg/ml have been reported [14]. These limitations make the mouse a suboptimal system for study of the effects of this protein. Nevertheless, significant effects of HRPII on BBB permeability and *P*. *berghei* pathogenesis were measured.

Given the long half-life of HRPII in the bloodstream of malaria parasites (it can be detected for more than a month after cured infection [16, 17]), our results raise the possibility that this protein may contribute to the persistent endothelial activation and inflammation seen after *P*. *falciparum* infection [18]. Whether this could cause lingering symptomatology remains to be established.

Blocking HRPII action has potential as an adjunctive therapy for falciparum malaria. Anti-IL-1β antibody was able to mitigate the effect of HRPII on vascular leakage in our mouse infusion studies. Drugs targeting IL-1β are already in clinical use, and these or other inhibitors that work elsewhere in the HRPII pro-inflammatory pathway could be considered for the treatment of cerebral malaria.

While the current work is focused on the brain, it will be interesting to see if other vascular beds are sensitive to HRPII, since falciparum malaria is a multi-organ disease. The long half-life of HRPII in the human bloodstream makes this molecule a candidate to explain the failure of antimalarial drugs to reverse cerebral malaria symptoms in some cases, even after parasite clearance. Similarly, it could offer an explanation for post-malaria syndrome symptomatology and for the persistent endothelial activation that has been observed after falciparum malaria has been treated.
